# Effect of 150 kHz electromagnetic radiation on the development of polycystic ovaries induced by estradiol Valerate in Sprague Dawley rats

**DOI:** 10.1186/s13048-021-00774-4

**Published:** 2021-02-05

**Authors:** Stephanie Mohammed, Venkatesan Sundaram, Nikolay Zyuzikov

**Affiliations:** 1 Department of Physics, Faculty of Science and Technology, The University of the West Indies, St. Augustine, Trinidad and Tobago; 2 Department of Basic Veterinary Sciences, School of Veterinary Medicine, Faculty of Medical Sciences, The University of the West Indies, St. Augustine, Trinidad and Tobago

**Keywords:** 150 kHz electromagnetic radiation, Estradiol Valerate, Polycystic ovary model

## Abstract

**Background:**

Polycystic ovary syndrome (PCOS) is the most common complex endocrine disorder affecting approximately 2–20% of reproductive aged females. Tumour Treating Fields (100–300 kHz) is a recent innovative, non-invasive therapeutic approach to cancer therapy. This frequency as an alternative therapy for the management of polycystic ovaries has not yet been explored.

**Objectives:**

To explore the effect of full-body exposure of 150 kHz Electromagnetic Radiation (EMR), on the development of polycystic ovaries in an estradiol valerate-induced PCO rat model.

**Method:**

Twenty-one female adult rats were divided into three groups (*n* = 7 each): control, Estradiol Valerate (EV) and EV + EMR groups. The EV + EMR group was subjected to full body exposure at 150 kHz EMR continuously for eight consecutive weeks. Estradiol valerate was administered orally to induce polycystic ovaries in EV and EV + EMR groups. Body and ovarian weights were recorded and analysed. The regularity of the estrous cycle was assessed in all three groups. The histological study of ovarian tissue was carried out by haematoxylin and eosin staining. The serum concentration levels of Luteinizing Hormone (LH), Follicle-Stimulating Hormone (FSH) and testosterone were measured using the ELISA method.

**Results:**

The body and ovary weights did not differ significantly between the EV and EV + EMR groups. The estrous cycle was found to be irregular in both the EV and EV + EMR groups. Ovarian histology revealed near normal morphology with little or no degenerative and morphological changes in developing follicles in the exposed group. Histometrical analysis showed an increased number of developing follicles and a significant reduction in the number and size of follicular cysts (*p* < 0.05) in the EV + EMR group. Hormonal analysis revealed no significant difference in the testosterone and FSH levels between the EV + EMR and EV groups. However, the LH, LH/FSH ratio decreased significantly in the EV + EMR group compares to the EV group.

**Conclusion:**

The 150 kHz EMR appear to have little or no degenerative and morphological changes in the developing follicles, an increased number of typical developing follicles and a significant reduction in the number and size of the follicular cysts (*p* < 0.05).

## Subject classification codes

Electromagnetic Radiation, Polycystic Ovary.

## Introduction

Polycystic ovary syndrome (PCOS) is recognized as the most common complex endocrine disorder affecting approximately 2–20% of reproductive aged females [8]. This condition manifests polycystic ovaries, hyperandrogenism, androgenic alopecia, hirsutism, acne, menstrual irregularity, anovulation or oligo-amenorrhea, miscarriage, and infertility [28]. It presents symptoms such as unwanted hair growth and hormonal changes which can negatively affect the emotional character of women which may subsequently may result in depression and anxiety [2, 15]. Women with PCOS are more susceptible to several chronic conditions including obesity, dyslipidaemia, hypertension, heart disease, and type 2 diabetes mellitus (T2DM) [20]. The definite aetiology of PCOS remains largely unknown. However, complex interactions between genetic, behavioural, and environmental factors play critical roles in the development of PCOS and subsequent therapeutic options [11]. Present treatment options focus on controlling the associated signs and symptoms. Therefore, the search for more efficacious, affordable treatment options attracts interest for the management of PCO and its subsequent complications.

Electromagnetic radiation (EMR) consists of electromagnetic waves, which are synchronized oscillations of both electric and magnetic fields that travel through a vacuum at the speed of light. These waves which are constantly emitted from the natural environment, as well as from everyday appliances, frequently influence the human body. The effect of this type of energy waves on living tissues may exert various effects on their ability to function, although the mechanisms conditioning this phenomenon have not been fully understood. The effects of the EMR on the reproductive system is categorized as hazardous, neutral or beneficial [34]. The results of reproductive studies confirming a beneficial effect of electromagnetic waves evoke hope for the need of these inventions in the treatment of PCO.

Currently, the Intermediate Frequency (300 Hz to 10 MHz) range has offered controversial outlook on the therapeutic use of this range of frequency. Confirmingly the range of 100 kHz – 300 kHz known as Tumor Treating Fields (TTF) has provided substantial evidence for a more positive advancement in the field of oncology. Tumor Treating Fields is an innovative, non-invasive and advanced therapeutic approach to various cancer therapies. This particular frequency disrupts mitosis and selectively kills rapidly dividing cancer cells by delivering continuous (over 18 h per day) low intensity, intermediate frequency, alternating electric fields (100 kHz – 300 kHz) to the tumor site [30]. Tumor Treating Fields have been found very effective in the treatment of several cancers including Glioblastoma multiforme and ovarian cancers [12, 17] in a preclinical setting.

It is postulated that the follicular disruption of PCOS is 2-fold [7]. First, the early follicular growth is extreme and second, the selection of one follicle from this increased pool of growing follicles for further maturation to a dominant follicle is arrested. It remains unknown whether the primary defects lie within the theca, granulosa or oocyte but it is presumed a consequence of intra-ovarian hyperandrogenism. Previous reports of TTFs on the action of abnormally proliferating cells, therefore evokes interest for exploring the effect of this frequency during the follicular development of polycystic ovaries.

The optimal frequency of TTFs for antimitotic effect varies by cancer type, and can be adjusted for maximal anticancer effect. In a preclinical setting, 150 kHz TTFs was found to be effective for pancreatic cancer, Non-Small-Cell lung carcinoma (NSCLC), brain metastasis from NSCLC and mesothelioma treatment when combined with chemotherapy [17]. Currently, 200 kHz is being explored for ovarian cancers in the same setting [33]. Therefore, this current study is designed to test the effect of 150 kHz Electromagnetic radiation (EMR) during the development of estradiaol valverate induced polycystic ovaries in rats.

## Materials and methods

### Animals and experimental design

A total of twenty-one (21) adult female Sprague Dawley (SD) rats (12–15 weeks-old) weighing 250-300 g were procured from the Lab Animal Facility at the School of Veterinary Medicine for the study. The animals were placed in ventilated metal cages with the dimensions of 40 × 24 × 14 cm (2 rats per cage) with paper bedding material in a pathogen free room at a temperature of 24 ± 2 °C, humidity of 50–60% and 12-h light/dark cycle. The rats were fed with a standard pellet diet and water ad libitum. The rats were allowed to acclimatize to laboratory condition for 7 days.

The animals were divided randomly into three groups (*n* = 7 each): control group, Estradiol Valerate Group (EV) and EV + EMR group. The EV + EMR group was subjected to full body exposure of EMR at 150 kHz continuously (except for about one hour per week that was needed for changing the cages) for eight consecutive weeks [1]. Polycystic ovarian condition were induced in the EV and EV + EMR groups of animals by administering commercially available estradiol valerate tablets at a single oral dosage of 4 mg per animal on the first and 14th day of experiment as reported by Brawer et al. [3] to ensure the EV activity was maintained for the development of PCO. The control and EV group was kept under similar conditions without EMR. The Campus Research Ethics Committee of the University of the West Indies approved the protocols for animal experimentation (CEC 310/09/17).

### Exposure device

The animals were kept in a uniform electromagnetic field with a frequency of 150 kHz and Amplitude voltage of 12 V. The electric signal was produced by Kenwood AG-203A oscillator 10 Hz-1 MHz with maximum outcome intensity. The field was generated by two parallel electrodes made of cardboard covered by aluminium foil. The electrodes were placed at opposite cage walls. The distance between electrodes of 40 cm was determined by the cage size, so, the amplitude field strength was 0.3 V/cm. The intensity of the field in the cages was measured by broadband (100 kHz-6 GHz) radiation meter Airmed Narda NBM-550. The control group of animals were in the same room and to reduce leaking radiation, the control and EV group cages were surrounded by foil on cardboards from all 4 sides. The intensity of field was 50–80 μW/cm^2^ inside experimental cages and 20–50 nW/cm^2^ in the control and EV cages. The overall room had an exposure of 0–100 nW/cm^2^. Thus the intensity of electromagnetic field in the irradiation cages was more than 1000 times higher in comparison to the control/sham exposed cages which was due to the generation of an EM field by the oscillator. During the electromagnetic field intensity measurements, all cellular devices were placed away. The device was the only source for emitting the desired EMR frequency. Geometry and positions of cages, electrodes and oscillator were not changed during the experiment. The EMR level was monitored weekly to ensure consistent levels of exposure to each cage and to each group.

### Assessment of estrous cycle

**Fig. 1:**
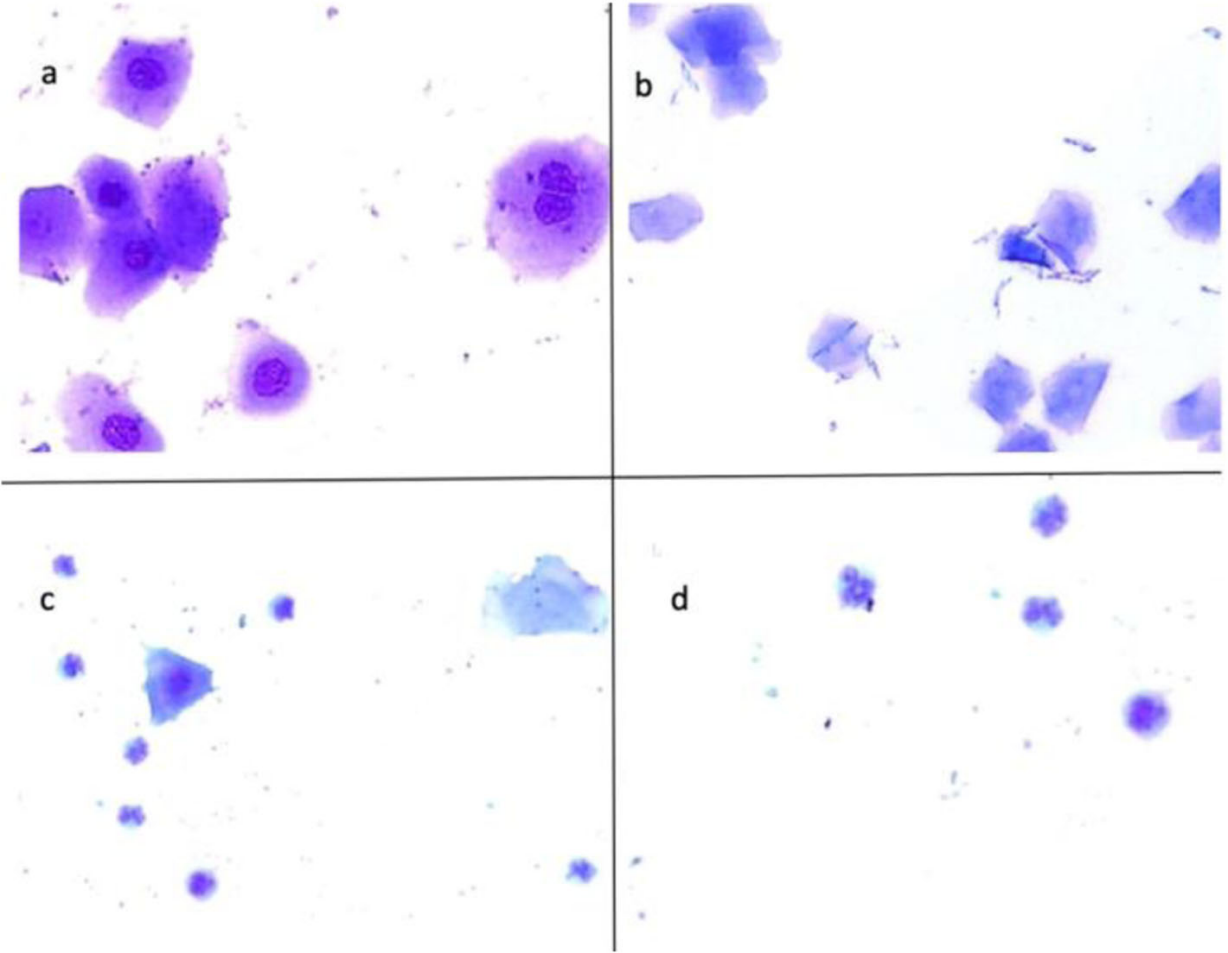
Exfoliative cytology during the estrous cycle. (**a**) Proestrous stage shows small nucleated cells. (**b**) Estrous stage shows cornified cells. (**c**) Metestrous stage shows nucleated, cornified and neutrophil cells. (**d**) Diestrous stage shows neutrophils

All animals were assessed for regularity of the estrous cycle by exfoliative vaginal cytology before and throughout the experiment. The animals with three consecutive normal estrous cycles alone were used for the study. The oestrus cycle was assessed by vaginal swab method. The vaginal smears were taken early in the morning daily at the same time to reduce variability and to ensure evaluators were aware of inherent variations. Cotton tipped swabs moistened with phosphate buffered saline were inserted into the vaginal cavity to obtain exfoliative cells. The cells were directly smeared onto clear microscope glass slides with pre-labelled identification numbers. The slides were immediately stained with Methylene blue and left to air dry [16]. After 10 min, vaginal cytology was analysed to determine the stage of the estrous cycle with the aid of an Olympus BX51 system microscope. The different stages of the estrous cycle were identified by exfoliative cytology as seen in (Fig. 1). These consisted of: Proestrous - predominance of small nucleated cells; Estrous - predominance of irregularly shaped epithelial cells with invisible nucleus; Metestrous - mixture of nucleated, cornified and neutrophils and Diestrous - predominance of neutrophils. The persistent vaginal cornification is a sign of PCO development and animals with these cytology is confirmed as PCO(S) animals.

### Hormonal analysis

At the end of the exposure period, the animals were weighed and sedated with ketamine hydrochloride at a dosage rate of 80 mg/kg intraperitoneally. Once the rats were sedated, they were put under deep anaesthesia by administering pentobarbital sodium at a dosage rate of 40 mg/kg intraperitoneally. Once the anaesthetic had taken effect, 5 ml of blood was collected using a standard terminal cardiac puncture protocol. Immediately after collection of blood, the animals were euthanized by overdosing with pentobarbital sodium at a dosage rate of 120 mg/kg intraperitoneally. The blood samples were centrifuged 1500 rpm for 10 mins at 4 °C and serum was separated. The serum samples were then stored at − 80 °C until testing. A testosterone ELISA kit (ab 108,666, Abcam), sensitive to 0.07 ng/ml, was used to measure the levels of testosterone in the serum. ELISA Assays for Luteinising Hormone (LH) (cat no. ENZKIT 107, Enzo Life Sciences) and Follicle Stimulating Hormone (FSH) (cat no. LS-F38636, Lifespan Biosciences, NC) were used to estimate levels of LH and FSH.

### Histological analysis

The ovaries were dissected out, weighed and fixed in 10% buffered neutral formalin and processed further by routine histological procedure. Sections were cut at 3–5 μm thickness using a rotary microtome (*Thermo Shandon Finesse ME*). The slides were stained with Haematoxylin and Eosin (H&E) using standard protocol and analysed with aid of the Olympus BX51 system microscope. All follicles were classified as either normal or atretic. Follicles with intact oocytes surrounded by layers of complete granulosa cells were considered as normal. While, atretic follicles presented with vacuolization and pyknotic nuclei within the granulosa cells and also some occasional shrinkage of oocytes. Photomicrographs were then taken with the help of an Olympus DP71 microscope digital camera.

### Histomorphometric analysis

The ovarian tissues that were stained with haematoxylin and eosin (H&E) were used for histomorphometry. Follicles were assigned four groups based on their developmental stage: (1) primordial follicles (oocytes of follicles surrounded by a layer of squamous or flattened granulosa cells); (2) primary follicles (oocytes surrounded by a single layer of cuboidal granulosa cells); (3) preantral/secondary follicles (oocytes surrounded by more than one layer of cuboidal granulosa cells with no antrum); and (4) antral follicles (oocytes surrounded by more than one layer of cuboidal granulosa cells with a visible antrum). A quantitative assessment was made by counting the number of follicles in each section of the ovary. Follicles with visible oocytes in the sections were counted three times and averaged [31]. The number of corpora lutea (CL) were also counted.

### Statistical analysis

Data was analysed with the use of IBM SPSS Statistics V21 (Armonk, New York, USA) software. Descriptive statistics were calculated for all animals used in the experiment. The mean and standard deviation were calculated among the categorical groups using ANOVA. Statistical significance was set at *p* < 0.05.

## Results

### Effect on body and ovary weight

The body weights measured at the end of the experiment revealed that the mean and standard deviation had reduced significantly when animals were given EV regardless of exposure. However, there was no significant difference among the EV group and the exposure group with regards to body weight. Additionally the weight of the left and right ovary did not vary significantly among all three groups (Table 1).

**Table 1:** The effect of 150 kHz EMR on the body and ovarian weights (*n* = 7)

Parameter	Control	EV	EV + EMR	p
Body weight/g	391.19 ± 51.28	283.27 ± 33.33	281.80 ± 23.46	0.14
Weight of left ovary/g	0.07 ± 0.02	0.08 ± 0.01	0.07 ± 0.03	0.74
Weight of right ovary/g	0.06 ± 0.02	0.07 ± 0.01	0.06 ± 0.02	0.40

*The mean difference is significant at the p = 0.05 confidence interval

### Effect on estrous cycle

**Fig. 2:**
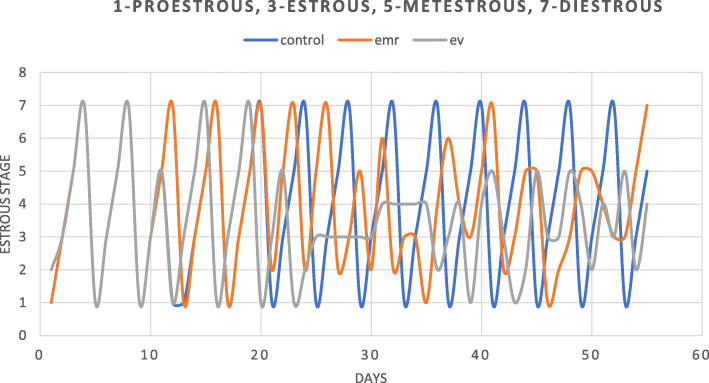
Representation of estrous cycle of EV and EV + EMR group for 46 days

All three groups showed normal estrous cycle prior to the experiment. During the experiment, the normal estrous cycle of 4–5 days with all four phases was observed in the control group, whereas it was disrupted in EV induced group with a dominant estrous stage (many cornified cells). The EV+ EMR showed less cornification stages with improved estrous cycle than the EV group (Fig. 2).

### Effect on histological structure of ovary

**Fig. 3:**
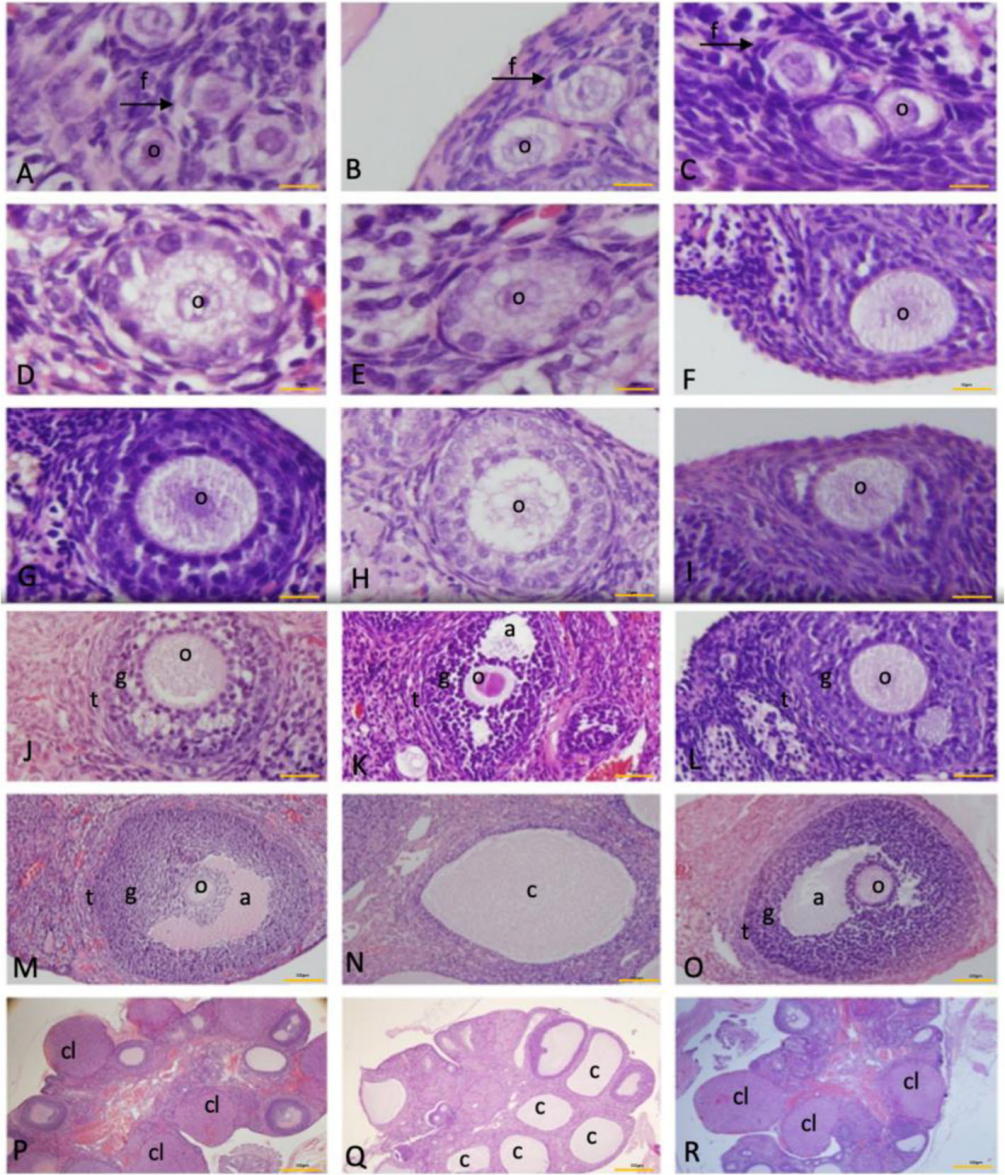
Photomicrographs of different ovarian follicles in control, EV and EV + EMR groups. The photomicrograph showing the nests of primordial follicles in the Control(**A**), EV(**b**) and EV + EMR (**c**) groups. The unilaminar primary follicles in the Control (**d**), EV (**e**) and EV + EMR (**f**) groups. The multilaminar primary follicles in the Control (**g**), EV (**h**) and EV + EMR (**i**) groups. The antral follicles in the Control (**j**), EV (**k**) with marked distorted granulosa and theca layer cells greater than the EV + EMR (**l**) groups. The matured follicle in the control (**m**) and EV + EMR (**o**) groups with less distortion of the granulosa cells. The cystic follicle (**n**) in the EV group with thin layer of granulosa cells. The cross section of the ovary of the Control (**p**), EV (**q**) and EV + EMR (**r**) groups. o- oocytes; f: follicular cells; g granulosa cells; t-thecal cells; a; a-antrum; c-cysts cl; corpus luteum gf; Graffian follicle

The ovarian follicles at different stages of development were normal and intact for the control group. The preantral and antral follicles revealed signs of degeneration, including cell pyknosis, thin granulosa cells layer, numerous cystic follicles, thickened theca layer, distorted zona pellucida and cumulus oophorous and blood congestion and reduced number of CL in EV group rats. The EV + EMR group showed little signs of distortion from the antral follicle to the mature follicle. Follicles at various stages were observed for this group (almost similar to the control), with a smaller number of cysts present (Fig. 3).

### Effect on histomorphometry of ovary

**Fig. 4:**
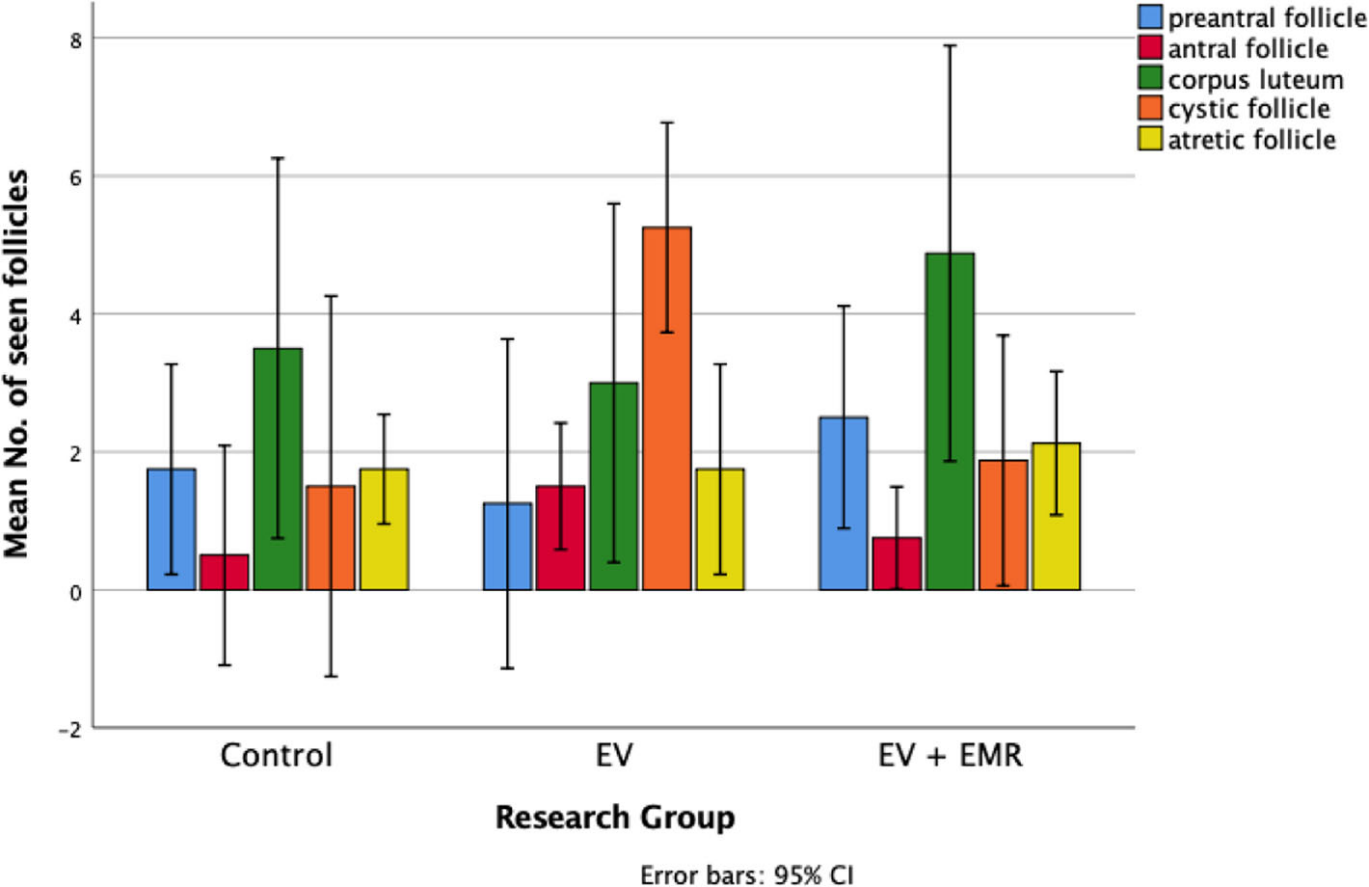
Effect of 150 kHz EMR in the follicular development in rats

**Fig. 5:**
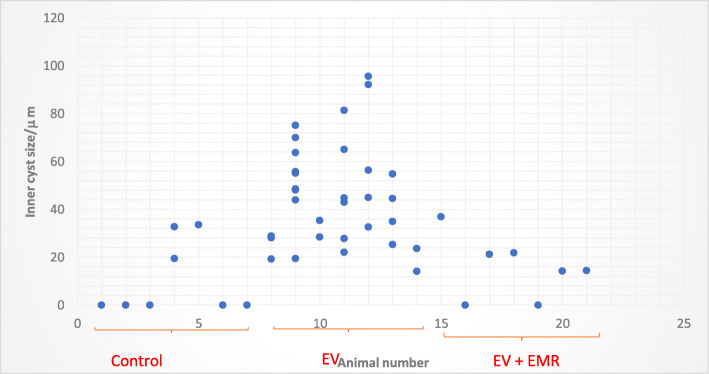
Effect of 150 kHz EMR on the distribution and size of follicular cysts in rats

The histomorphometric analysis of ovarian follicles in the control, EV and EV + EMR groups are presented in (Fig. 4). In the EV group, a significant decline was observed in the number of preandral follicles whereas, the number of antral follicle and cystic follicle increased in number compared with control and EV + EMR groups. The number of atretic follicles did not show any significant difference among the groups. In EV + EMR group, the number of ovarian follicles at the different stages of development were closely similar to the control group. The number of corpus luteum was lower in the EV group and highest in the EV + EMR among all three groups. The mode number of follicular cysts per ovary in the EV group was higher than all groups as each rats presented with at least 2 cysts with inner diameter > 40 μm. Two rats in the control group were observed to have cysts with inner diameter < 40 μm as seen in (Fig. 5). The EV + EMR group had an average of 1 follicular cyst per animal with inner diameter < 30 μm and some had no visible follicular cysts.

### Effect on gonadotrophic hormones

**Fig. 6:**
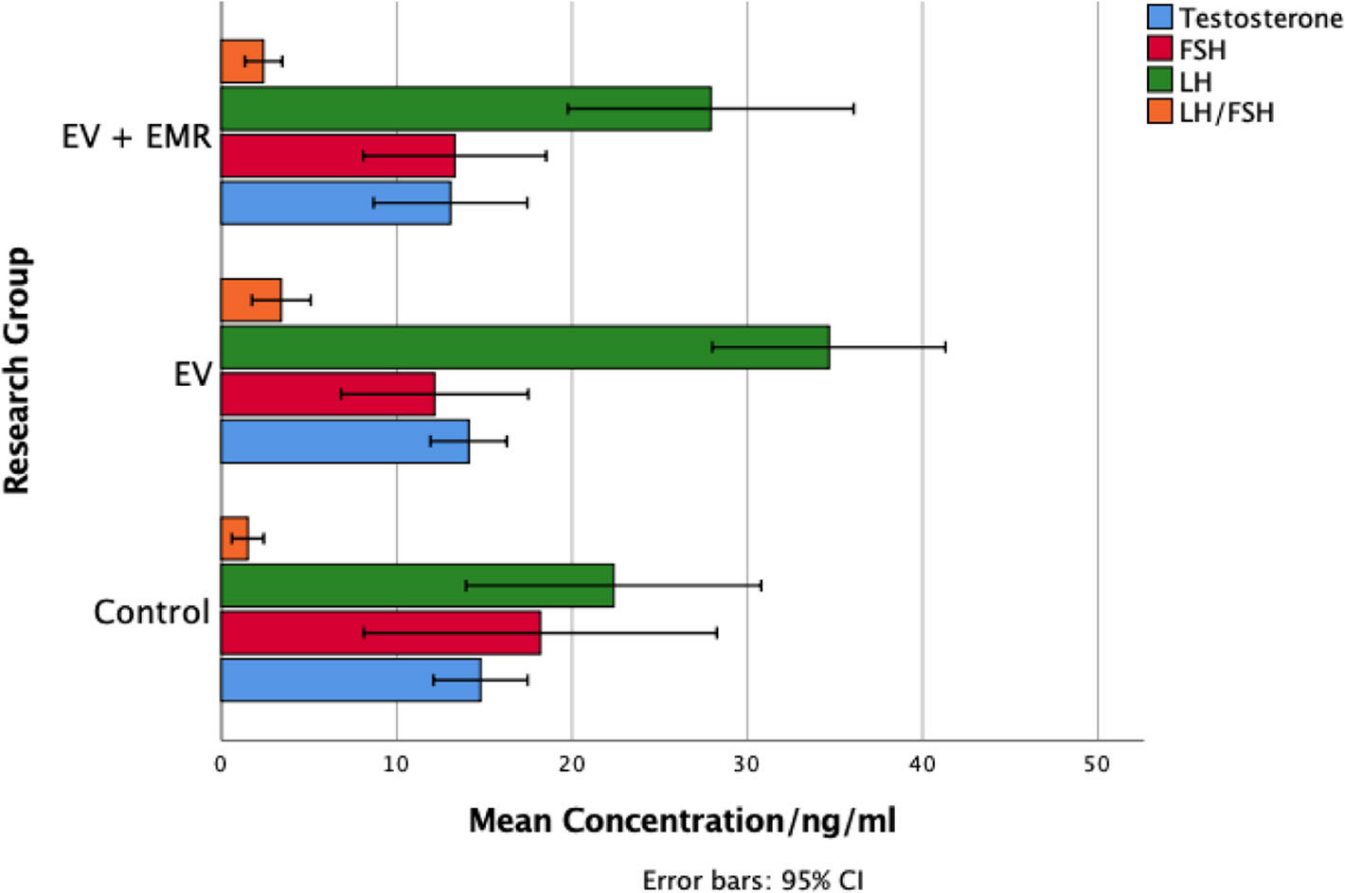
Effect of 150 kHz EMR on serum levels of gonadotrophic hormones in the rats

The serum concentrations of gonadotropic and sex hormones in the control, EV and EV + EMR are presented in (Fig. 6). There was a significant difference in the LH levels between control (22.37 ± 9.10 ng/ml), EV (34.66 ± 7.19 ng/ml) and EV + EMR (27.92 ± 8.82 ng/ml) with *p* = 0.04 with an increase in EV group. The LH/FSH ratio was also significantly (*p* = 0.05) different among the groups (*control –* times 1; *EV –* times 3 and *EV + EV&EMR –* times 2). The control group (18.19 ± 10.90 ng/ml) showed the highest level of FSH when compared to the EV group (12.16 ± 5.77 ng/ml) and the EV + EMR group (13.30 ± 5.65 ng/ml). There was no significant difference in the testosterone levels among the three groups as *p* = 0.66.

## Discussion

The present study successfully induced the pathophysiological development of PCOS as identified in humans [5]. Estradial valerate chemically designated as estra-1, 3, 5(10)-triene-3,17β-diol 17β-pentanoate, a synthetic sterane steroid, long- acting oestrogen on administration caused hypothalamic–pituitary dysregulation of GnRH, resulting in improper release and storage of LH [3, 4]. The young cyclic adult rats treated with single dose of 4 mg of EV per animal on 1st and 14th day of [3] experiment in the present study developed a successful polycystic ovary which was confirmed by the presence of numerous large follicular cysts without an oocyte, reduced granulosa cells, theca layer hyperplasia and reduced number of corpus lutea, very much similar to women PCOS [27]. The vaginal exfoliative cytology, a key indicator of ovarian physiology, confirmed that EV treated rats in the present study were almost acyclic, specifying the presence of cysts contrary to the control group similar to that reported by [10].

The EV + EMR exposed groups in the present study exhibited several positive effects such as, slightly lower body weight but not by a significant amount, improved reproductive cycle, usual morphology of developing follicles, increased number of typical developing follicles, reduction in the mean number and diameter of the follicular cysts than the PCOS rats (EV group) and closely similar to the control group. The most important finding in the study is the reduction in the number of follicular cysts present per animal. The highest number of cysts in the EV + EMR group was only two cysts per animal and some had none. This revealed that the EMR might have reduced the formation of the cysts.

The present study revealed that overall follicular dynamics was less disrupted in the group exposed to EMR and the observations were very close to the control group. This is because the follicular developmental staging from primordial to secondary was observed to be present. The follicular cell differentiation into granulosa cells and thecal cells with little to no distortion and vacuolisation when compared with EV group was also evident. However, a lot of research has focused on the harmful effects of EMFs on the granulosa cells of the oocytes. Apoptosis of these cells was reported in many articles due to EMFs ([26]. An increase in the number of macrophages and autophagy vacuoles in granulosa cell layers were identifiable with transmission electron microscopy from research conducted on the effects of EMFs on female rats. The study also revealed liquid drops in the luteal and theca cells [25].

Another research showed an increase in macrophages in the corpora lutea and growing follicles with EMFs exposure. Assumption is made that the process of apoptosis in the ovaries is increased with EMFs exposure. The destruction of ovarian cortical tissue, luminal epithelium, glandular epithelium, and stromal cells in the uterus and fallopian tubes are believed to result from the process of apoptosis from EMF exposure [22]. However, the 150 kHz EMR exposure in the present study did not show any of the above changes in the ovarian tissue.

The hormone progesterone is produced by the corpus luteum. This hormone is responsible for the control of the reproductive cycle and in return the preparation of the uterus for implantation if conception happens [29]. The reduced number of corpus luteum demonstrated an anovulatory state in the EV group that makes chances of conception minimal [21]. The EV + EMR exposed group showed a higher number of corpus lutea than the EV group and the reproductive cycle was an improved one in this group than the EV group. Morphological atresia was evident in all three groups. No major comparison was observed as follicular atresia is considered an active cellular process. Yet, the susceptibility to programmed cell death at various stages during follicular development remains undefined [26].

A key factor in hormone function changes and causes of infertility symptoms in females are the result of neuroendocrine changes caused by the impact of EMFs on females [19]. The decrease in number preandral follicles in the PCOS ovaries cause the overproduction of androgens that impedes with normal follicular maturation process [24]. But the present study did not show significant elevation in the testosterone level among the all groups. In the present study, the FSH concentrations did not alter but LH concentrations increased in rats with PCOS, thus the maturation of follicles was impaired and multi-sized cystic follicles were formed. The LH/FSH ratio in EV group and EV + EMR group were also significantly higher than the control group. The group exposed to EMR showed an increase in the LH and LH/FSH ratio, which is contrary to the reports in a DHEA-induced PCOS rat model [9, 23]. Generally, a high frequency in gonadotropin-releasing hormone (GnRH) pulses in the hypothalamus leads to LH secretion from the pituitary. In this case, increased levels can also be from accelerated GnRH activity, increased responsiveness to GnRH or decreased sensitivity of the hypothalamus received via negative feedback from sex steroids [32].

Overall, the reduction in cystic formation from exposure to NIR can be a possible avenue for further research. The possible mechanism on which this 150 kHz works can be linked to some of the Bio-Electromagnetic Principles. One major principle being the sensitivity of receptor efficiency on the surface of target cells to signal transduction [18] but cannot be confirmed in this experiment.

As alternating electric fields of intermediate frequency and low intensity, the TTFields have been reported to slow down the growth of tumor cells while having no obvious bioeffects on normal cells [6, 13, 30]. However, this frequency has never been examined during the development of non-cancerous conditions such as PCO. Hence, the consistency of the present results cannot be confirmed without results of the mechanistic studies involved in cancer cell lines in this frequency.

The effect of EMR on cells can be direct as shown by previous experiments on glioma cell lines [14] but it is unlikely because the same changes in all different layers of different types of cells in ovaries were observed in the present study. It can be speculated that there is an indirect effect either by the influence of EMR to cell receptors or the effect on the hypothalamus and signalling via certain hormones. Since the present study is focussed on the effect of 150 kHz on estrous cycle, ovarian histology and serum levels of gonadotrophic hormones, which is not strong enough to come to a solid conclusion. So, further investigations are required to assess the effect of EMR during follicuogenesis of PCO development by investigating the follicular ultrastructure and immunohistochemical characterisation of surface receptors of the granulosa and thecal cells and a detailed study on the Hypothalamo-hypopysio-gonal axis which forms the limitation of this study. Additionally, the study should be explored using various PCO inducing models to evaluate a more definite conclusion on cystic development. The authors are currently repeating this experiment to fully understand the effect of 150 kHz EMR on the HPG axis.

## Conclusion

The 150 kHz EMR appears to have a positive effect like improved reproductive cycle, reversal to usual morphology of developing follicles, increased number of typical developing follicles, reduction in the mean number and diameter of the follicular cysts. However, a more detailed study, which includes the limitations as highlighted.

## Data Availability

All data is available for this experiment. It will not be released because there are other phases of this experiment in progress.

## Abbreviations

CL: Corpora lutea
DHEA: Dehydroepiandrosterone
ELISA: Enzyme-linked Immunosorbent Assay
EM: Electromagnetic
EMF: Electromagnetic Frequency
EMR: Electromagnetic Radiation
EV: Estradiol Valverate
FSH: Follicle Stimulating Hormone
GnRH: Gonadotropin-releasing hormone
H & E: Haematoxylin and Eosin
LH: Luteinizing Hormone
NIR: Non-ionizing Radiation
PCO: Polycystic ovary
PCOS: Polycystic Ovary Syndrome
SD: Sprague Dawley
